# Ethanol Exposure Causes Muscle Degeneration in Zebrafish

**DOI:** 10.3390/jdb6010007

**Published:** 2018-03-09

**Authors:** Elizabeth C. Coffey, Maggie E. Pasquarella, Michelle F. Goody, Clarissa A. Henry

**Affiliations:** 1 School of Biology and Ecology, University of Maine, Orono, ME 04469, USA; elizabeth.c.mason@maine.edu (E.C.C.); margaret.pasquarella@maine.edu (M.E.P.); Michelle.Goody@maine.edu (M.F.G.); 2 Graduate School of Biomedical Sciences and Engineering, University of Maine, Orono, ME 04469, USA

**Keywords:** zebrafish, muscle, ethanol, alcohol, myopathy, fetal alcohol spectrum disorder, muscular dystrophy, Paxillin

## Abstract

Alcoholic myopathies are characterized by neuromusculoskeletal symptoms such as compromised movement and weakness. Although these symptoms have been attributed to neurological damage, EtOH may also target skeletal muscle. EtOH exposure during zebrafish primary muscle development or adulthood results in smaller muscle fibers. However, the effects of EtOH exposure on skeletal muscle during the growth period that follows primary muscle development are not well understood. We determined the effects of EtOH exposure on muscle during this phase of development. Strikingly, muscle fibers at this stage are acutely sensitive to EtOH treatment: EtOH induces muscle degeneration. The severity of EtOH-induced muscle damage varies but muscle becomes more refractory to EtOH as muscle develops. NF-kB induction in muscle indicates that EtOH triggers a pro-inflammatory response. EtOH-induced muscle damage is p53-independent. Uptake of Evans blue dye shows that EtOH treatment causes sarcolemmal instability before muscle fiber detachment. Dystrophin-null *sapje* mutant zebrafish also exhibit sarcolemmal instability. We tested whether Trichostatin A (TSA), which reduces muscle degeneration in *sapje* mutants, would affect EtOH-treated zebrafish. We found that TSA and EtOH are a lethal combination. EtOH does, however, exacerbate muscle degeneration in *sapje* mutants. EtOH also disrupts adhesion of muscle fibers to their extracellular matrix at the myotendinous junction: some detached muscle fibers retain beta-Dystroglycan indicating failure of muscle end attachments. Overexpression of Paxillin, which reduces muscle degeneration in zebrafish deficient for beta-Dystroglycan, is not sufficient to rescue degeneration. Taken together, our results suggest that EtOH exposure has pleiotropic deleterious effects on skeletal muscle.

## 1. Introduction

Ethanol (EtOH) is a teratogen that can cause multisystemic diseases at any stage of life. The rate of lifetime alcohol use disorders is approximately 12% in the US [1]. Chronic exposure in adults (alcoholism) can impair motor skills and cause fatigue, pain, and cramping [2]. These symptoms are similar to neuropathy [3]. Children with fetal alcohol spectrum disorders (FASD), which arise from maternal consumption of alcohol during pregnancy, exhibit compromised motor skills and full body weakness [4]. These symptoms are also attributed to neurological defects. FASD occurs in about 1 out of every 1000 live births [5]. In addition to the effect of EtOH on the nervous system, data indicate that skeletal muscle may also be affected by EtOH. Alcoholism in adults can cause a 30% reduction in muscle mass [3]. Early life stage exposure to EtOH causes skeletal muscle necrosis in chick embryos [6], decreases both fiber number and fiber size in rat pups born to EtOH-treated mothers [7], and decreases fiber size in zebrafish embryos exposed to EtOH during primary muscle development [8]. Adult zebrafish exposed to EtOH have decreased muscle fiber size and exhibit abnormal behavior that varies between strains [9,10]. The effects of EtOH exposure between primary muscle development and adulthood are not understood. 

Zebrafish are an ideal model for studying the effects of EtOH exposure on skeletal muscle after primary muscle development. Zebrafish skeletal muscle is structurally similar to that of other vertebrates, with individual muscle fibers and segmentally reiterated groups of muscle fibers surrounded by distinct and dynamic regions of extracellular matrix (ECM) [11,12,13,14,15,16,17]. An important substructure in the muscle ECM is the myotendinous junction (MTJ). The MTJ serves as the site of force transmission from muscle to bone and is conserved in fish and mammals [18]. The Dystrophin-glycoprotein complex (DGC) is one adhesion complex that anchors muscle fibers to laminin at the MTJ [19]. Dystrophin is the DGC component that provides a critical link between the muscle cell membrane (sarcolemma) and the cytoskeleton. Duchenne/Becker muscular dystrophy (DMD), results from mutations in *dystrophin* [20]. Mutations in the *laminin a2* gene cause congenital muscular dystrophy with merosin deficiency [21]. Despite the fact that these two muscular dystrophies result from mutations in components within the same adhesion complex, zebrafish and mammalian models for these two diseases showed that the cellular pathology underlying their muscle degeneration is different [22,23,24]. In the zebrafish model of DMD, damage to the sarcolemma is observed before muscle fibers detach from the ECM at the MTJ [24]. In contrast, muscle fibers in the zebrafish model of merosin-deficient congenital muscular dystrophy detach from the MTJ in the absence of sarcolemmal damage [24]. Thus, the primary cause of muscle degeneration in dystrophin-null *sapje* mutant zebrafish is sarcolemmal damage. In contrast, the primary cause of muscle damage in *laminin a2* mutant zebrafish is failure of adhesion of muscle fibers to the MTJ. We found that human influenza A virus causes both sarcolemmal damage and disrupts muscle fiber adhesion to the MTJ [25], indicating that these modes of damage are not mutually exclusive. 

In this study, we show that EtOH treatment after primary muscle development disrupts muscle homeostasis and causes muscle degeneration. Similar to previous reports [8,9], the penetrance and severity of EtOH-induced muscle damage was somewhat variable. Despite this variability, the severity of muscle damage caused by EtOH exposure significantly decreased by 48 hpf, suggesting that muscle becomes more refractory to EtOH exposure as development proceeds. EtOH treatment caused sarcolemmal damage and disrupted adhesion of muscle fibers to the MTJ. Interestingly, neither a treatment that improves pathology in zebrafish with sarcolemmal damage (Trichostatin A (TSA) treatment) nor a treatment that improves pathology in zebrafish with disrupted muscle-MTJ adhesion (Paxillin overexpression) was beneficial in EtOH-exposed zebrafish. EtOH treatment triggered a robust, pro-inflammatory cellular response as indicated by aberrant activation of NF-kB muscle. Taken together, these data suggest that EtOH exposure induces cellular stress, sarcolemmal damage, and disrupts muscle fiber-MTJ adhesion.

## 2. Materials and Methods

### 2.1. Zebrafish Husbandry, Transgenic Lines, and Mutant Lines

Zebrafish embryos were retrieved from natural spawns of adult zebrafish maintained on a 14-h light/10-h dark cycle. Strains used in this study were AB, *Tg(actb2:pxn-EGFP)^mai1^* [26], *Tg(hsp70l:pxn-EGFP)*, *Tg(Nf-kB:EGFP)^nc1^* [27], *tp53^M214K^* [28], and *sapje^ta222a^* [29]. Embryos were grown in embryo rearing media (1X ERM) with methylene blue at 28.5 or 33 degrees Celsius and staged according to [30]. All protocols conformed to the University of Maine Institutional Animal Care and Usage Committee’s Guidelines that do not require permission for the use of zebrafish 3 days old and younger.

### 2.2. Ethanol (EtOH) and Trichostatin A (TSA) Treatment

EtOH dilutions were chosen based on [8]. Solutions of 2% EtOH diluted in 1X ERM from either 95% or 100% EtOH stocks were continuously administered beginning at different stages of development (24, 30, 36, or 48 hpf) through 72 hpf. Treatments with the histone deacetylase (HDAC) inhibitor TSA were carried out according to [31]. TSA was dissolved in either DMSO or EtOH. TSA (200 nM) was diluted in 1X ERM and administered continuously from 24 to 72 hpf alone or from 24 to 30 hpf alone and then concurrently with 2% EtOH from 30 to 72 hpf. Embryos were dechorionated prior to EtOH or TSA administration. All solutions were changed daily.

### 2.3. Heat Shock Treatment

Heat shock was done at 6 or 24 hpf in a 37–38 degrees Celsius incubator for 1–2 h. All heat shock-treated embryos were screened using a GFP 470 filter on a Zeiss Discovery.V12 microscope to identify fluorescent transgenic embryos.

### 2.4. Evans Blue Dye (EBD) Injection

Zebrafish were anesthetized in 0.16 mg/mL tricaine in 1X ERM and side mounted on 1% agarose-lined Petri dishes in a minimal volume of liquid. EBD (1% *w*/*v*) was dissolved in a 0.9% saline solution. Injection needles were pulled from glass capillary tubes containing a filament on a Sutter Flaming/Brown Micropipette Puller. EBD solution was loaded into injection needles and injected into the circulation of anesthetized ~72 hpf zebrafish using a MPPI-3 pressure injector from ASI. EBD injected zebrafish were maintained in 1X ERM for 6 h before live imaging.

### 2.5. Immunohistochemistry

Zebrafish embryo fixation, phalloidin staining, and beta-Dystroglycan immunohistochemistry was done according to [32].

### 2.6. Imaging

Images were taken on a Zeiss Axio Imager Z1 microscope with a Zeiss ApoTome attachment or an Olympus Fluoview IX-81 inverted microscope with FV1000 confocal system. Embryos were side mounted in 80% glycerol/20% PBS and viewed with the 20× objective. Exposure times were kept constant throughout the imaging of an experiment allowing for the comparison of fluorescent levels between images. Live imaging for EBD experiments involved side mounting ~78 hpf zebrafish embryos in 0.4% low melt agarose containing 0.16 mg/mL tricaine and imaging via confocal microscopy. Linear adjustments were made to images in Adobe Photoshop and figures were collated in Adobe Illustrator.

### 2.7. Analysis and Statistics

Statistical comparisons of two groups were conducted with student t-tests. Statistical comparisons of multiple groups were determined using ANOVA followed by Tukey’s honest significant difference multiple comparisons test. For ANOVA details, please see the supplementary materials. Statistical analysis was done using Prism 7 or Microsoft Excel.

## 3. Results

### 3.1. Late Embryonic Exposure to EtOH Causes Muscle Damage in Zebrafish

**Figure 1:**
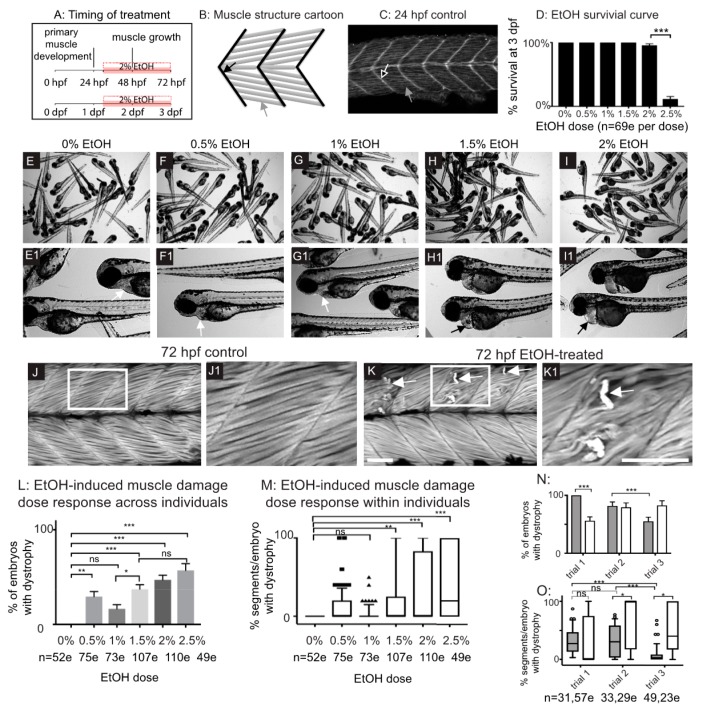
Late embryonic exposure to EtOH causes muscle damage in zebrafish. (**A**) Zebrafish muscle development timeline showing that 2% EtOH was administered after primary muscle development is complete. (**B**) Cartoon showing zebrafish muscle structure. Elongated myofibers (gray arrow) attach at the myotendinous junction (MTJ) (black arrow). (**C**) Control embryo. Muscle fibers have elongated (gray arrow) and attached to the MTJ (black arrow outlined in white) by 24 hpf. (**D**) Quantification of survival at 72 hpf following continuous treatment with one of six different doses of EtOH from 30 to 72 hpf. (**E**–**I1**) Brightfield images of 72 hpf zebrafish treated with one of six different doses of EtOH. Numbered panels are higher magnification images of zebrafish treated with the same EtOH dose as the corresponding lettered panels. (**E**–**E1**) 0% EtOH. Note normal heart morphology (**E1**, white arrow). (**F**–**F1**) 0.5% EtOH. Note normal heart morphology (**F1**, white arrow). (**G**–**G1**) 1% EtOH. Note normal heart morphology (**G1**, white arrow). (**H**–**H1**) 1.5% EtOH. Note pericardial edema (**H1**, black arrow). (**I**–**I1**) 2% EtOH. Note pericardial edema (**I1**, black arrow). (**J**–**K1**) Anterior left, dorsal-top, side-mounted, 72 hpf embryos stained with phalloidin (white) to visualize actin. White boxes in **J** and **K** correspond to zoomed-in, numbered panels **J1** and **K1**, respectively. (**J**–**J1**) Control embryo. Muscle fibers remain attached to the MTJ. (**K**–**K1**) Embryo treated continuously with 2% EtOH from 30 to 72 hpf. White arrows indicate muscle fibers that have detached from the MTJ. (**L**) Quantification of the percent of EtOH-treated embryos at each of the EtOH treatment doses that displayed one or more fiber detachments. (**M**) Quantification of the percent of imaged segments with fiber detachments per embryo across the six different EtOH treatment doses. (**N**–**O**) Quantification of the variability in EtOH-induced fiber detachments across experimental replicates. (**N**) Percent of EtOH-treated embryos with at least one fiber detachment across three trials with either 2% EtOH diluted from 95% stock (gray bars) or 2% EtOH diluted from 100% stock (white bars). (**O**) Percent of imaged segments per embryo with fiber detachments across three trials with either 2% EtOH diluted from 95% stock (gray boxes) or 2% EtOH diluted from 100% stock (white boxes). Note the persistence of variability in muscle damage across three trials regardless of EtOH stock solution used. Scale bars are 50 micrometers. “e” = embryo. Error bars are standard error of the mean and whiskers are 10th–90th percentiles. * *p* < 0.05, ** *p* < 0.01, *** *p* < 0.001.

Zebrafish primary muscle development entails the elongation of short muscle precursor cells into long myofibers that attach to the ECM at the MTJ and undergo fusion to generate myotubes [33]. This process occurs by 24 post fertilization (hpf) (Figure 1A–C). Exposure to 2 or 2.5% EtOH during primary muscle development (8–24 hpf) results in smaller muscle fibers and abnormally wide MTJs [8]. We asked if muscle integrity would be disrupted in zebrafish that were treated with EtOH after primary muscle development during a period of muscle growth (Figure 1A). We first assessed survival and found that treatment with 0–2% EtOH did not drastically affect survival at 72 hpf (Figure 1D). In contrast, most zebrafish treated with 2.5% EtOH died by 72 hpf (Figure 1D). Exposure to lower concentrations of EtOH (0.5–1%) did not cause gross morphological phenotypes (Figure 1E–G1). Exposure to 1.5% EtOH or higher resulted in fully penetrant pericardial edema (Figure 1H–I1 *n* = 52/52 and 53/53 for 1.5 and 2% EtOH, respectively). EtOH exposure also caused muscle damage. Control zebrafish had linear, organized muscle fibers at 24 hpf (Figure 1C, white arrow) and at 3 dpf (Figure 1J–J1). EtOH treatment caused muscle fiber degeneration (Figure 1K–K1, white arrows) although there was not a clear dose-dependent effect (Figure S1L,M, supplementary materials). These data suggest that EtOH negatively affects skeletal muscle homeostasis in zebrafish.

Although treatment with 2% EtOH diluted from a 95% (190 proof) stock resulted in fiber detachment, the penetrance of this phenotype varied across experimental replicates (Figure 1N,O). We also treated zebrafish with EtOH diluted from a 100% (200 proof) stock and observed the same fiber detachment phenotype with similar variability in phenotypic frequency between experimental replicates (Figure 1N,O). Therefore, the variable penetrance of the EtOH-induced skeletal muscle phenotype is independent of pericardial edema and the stock solution used. All subsequent EtOH dilutions were made from the 95% stock. These data indicate that EtOH treatment after primary muscle development results in muscle degeneration.

### 3.2. Zebrafish Muscle Is Less Sensitive to EtOH That Is Administered Later in Development

**Figure 2:**
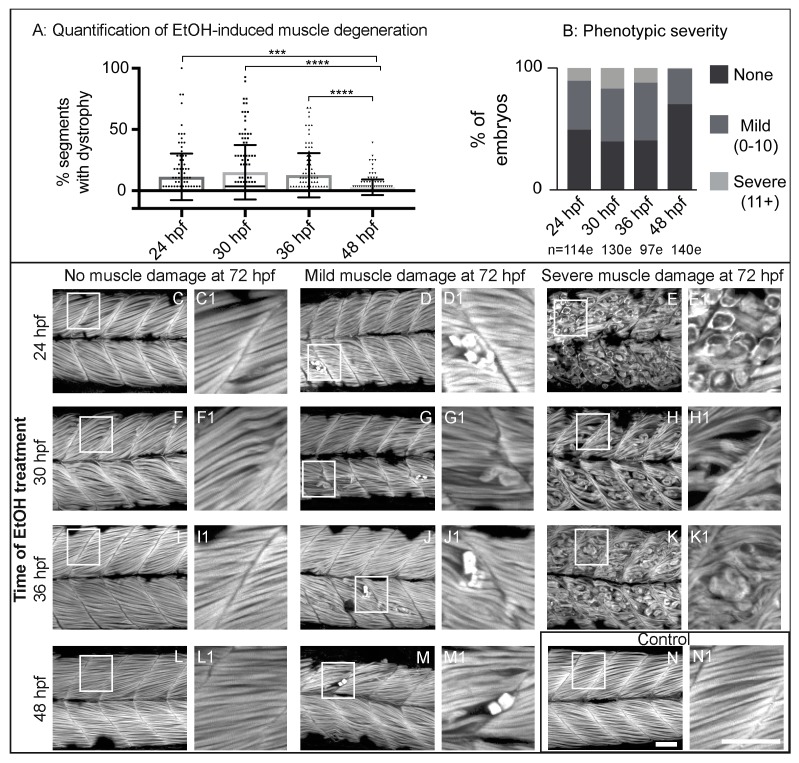
Zebrafish muscle is less sensitive to EtOH administered later in development. (**A**) Quantification of the percent of imaged segments with fiber detachments per embryo. 2% EtOH treatment from 48 to 72 hpf caused significantly fewer segments with fiber detachments per embryo compared to EtOH treatment beginning at 24, 30, or 36 hpf. (**B**) Quantification of the percent of embryos at each of the four EtOH treatment time points that fell into each of the three phenotype bins. (**C**–**N**) Anterior left, dorsal top, side-mounted, 72 hpf embryos stained with phalloidin (white) to visualize actin. Embryos treated continuously with 2% EtOH from 24 hpf, 30 hpf, 36 hpf, or 48 hpf through 72 hpf showed a spectrum of muscle damage severity that was binned into one of three groups: None (0 segments per embryo with dystrophy), Mild (1–10 segments per embryo with dystrophy), or Severe (11+ segments per embryo with dystrophy). White boxes correspond to zoomed-in, numbered panels. (**C**–**E1**) Embryos continuously treated with 2% EtOH between 24 and 72 hpf showed no muscle damage (**C**–**C1**), mild damage (**D**–**D1**), or severe damage (**E**–**E1**). (**F**–**H1**) Embryos continuously treated with 2% EtOH between 30 and 72 hpf showed no muscle damage (**F**–**F1**), mild damage (**G**–**G1**), or severe damage (**H**–**H1**). (**I**–**K1**) Embryos continuously treated with 2% EtOH between 36 and 72 hpf showed no muscle damage (**I**–**I1**), mild damage (**J**–**J1**), or severe damage (**K**–**K1**). (**L**–**M1**) Embryos continuously treated with 2% EtOH between 48 and 72 hpf showed no muscle damage (**L**–**L1**) or mild damage (**M**–**M1**). (**N**–**N1**) Control embryo. Scale bars are 50 micrometers. “e” = embryo. Error bars are standard error of the mean. *** *p* < 0.001, **** *p* < 0.0001.

Zebrafish primary muscle development is followed by a period of growth [34,35]. Less obvious, but significant changes also occur in the ECM at the MTJ during this time (reviewed in [36]). Thus, we asked whether there were any critical time windows in which EtOH administration would result in increased muscle damage. We treated embryos continuously with 2% EtOH beginning at 24, 30, 36, or 48 hpf and fixed them at 3 dpf. We found a spectrum of muscle damage severity following EtOH administration at each time point. However, muscle phenotypes were significantly less severe when zebrafish were EtOH-treated at 48 hpf (Figure 2A). To better analyze the muscle damage spectrum, we binned fiber detachment phenotypes into one of three groups: none (0 muscle segments with fiber detachments), mild (1–10 segments with fiber detachments), or severe (11+ segments with fiber detachments). Zebrafish treated with EtOH beginning at 24, 30, or 36 hpf displayed the full spectrum of muscle damage severity (Figure 2B–K1). In contrast, treatment at 48 hpf resulted in either no or only mild muscle damage (Figure 2B,L–M1). These data indicate that zebrafish are more refractory to EtOH-induced muscle damage later in development.

### 3.3. p53-Dependent Apoptosis Is Not Required for EtOH-Induced Fiber Detachments

**Figure 3:**
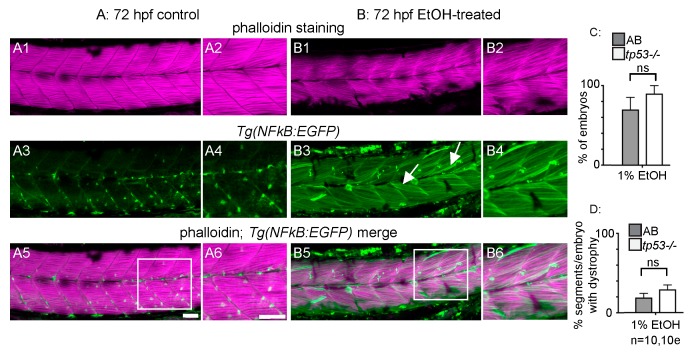
NF-kB-dependent inflammation is present, but p53-dependent apoptosis is not required for EtOH-induced fiber detachments. (**A1**–**B6**) Anterior left, dorsal top, side-mounted 72 hpf *Tg(NF-kB:EGFP)* embryos stained with phalloidin (pseudo-colored fuchsia) to visualize actin. (**A1**–**6**) 72 hpf *Tg(NF-kB:EGFP)* control embryo. (**A1**–**2**) Phalloidin channel only. (**A2**) Zoomed-in view corresponds to white box in A5. (**A3**–**4**) GFP channel only. (**A4**) Zoomed-in panel corresponds to white box in A5. Note low NF-kB activity in muscle fibers. (**A5**) Phalloidin and NF-kB-EGFP channels merged. (A6) Zoomed-in panel corresponds to white box in A5. (**B1**–**6**) 72 hpf *Tg(NF-kB:EGFP)* embryo treated continuously with 2% EtOH from 30 to 72 hpf. (**B1**–**2**) Phalloidin channel only. (**B2**) Zoomed-in view corresponds to white box in B5. (**B3**–**4**) GFP channel only. (**B4**) Zoomed-in view corresponds to white box in B5. Note increased NF-kB activity in muscle fibers due to EtOH exposure. White arrows points to muscle fibers expressing GFP. (**B5**–**6**) Phalloidin and NF-kB-EGFP channels merged. (**B6**) Zoomed-in view corresponds to white box in B5. (**C**–**D**) Quantification of fiber detachment frequency within and between AB or *tp53* mutant embryos treated with EtOH from 30 to 72 hpf. (**C**) Percent of EtOH-treated embryos displaying one or more fiber detachments. There was no significant difference in fiber detachment frequency between embryos depending on AB (gray bars) vs. *tp53* mutant (white bars) genetic background at 1% EtOH. (**D**) Percent of imaged segments with fiber detachments per embryo. There was no significant difference in fiber detachment frequency within embryos depending on AB (gray bars) vs. *tp53* mutant (white bars) genetic background at 1% EtOH. Scale bars are 50 micrometers. “e” = embryo. Error bars are standard error of the mean.

To begin characterizing the muscle damage caused by EtOH treatment, we first investigated whether inflammation or apoptosis may be involved. Activation of the transcription factor NF-kB is part of a pro-inflammatory, cellular stress response that can result in tissue damage. We used the *Tg(NF-kB:EGFP)* [27] zebrafish line to ask whether NF-kB-dependent signaling is altered upon EtOH exposure. *Tg(NF-kB:EGFP)* zebrafish were continuously exposed to 0 or 2% EtOH from 30 hpf on and then fixed at 3 dpf. Muscle fibers in control *Tg(NF-kB:EGFP)* zebrafish were long and linear (Figure 3A1,A2) and did not express GFP at 72 hpf (Figure 3A3–A6, [25]). EtOH treatment increased NF-kB activity as evidenced by increased GFP expression in muscle fibers (Figure 3B3–B6). Therefore, muscle cells respond to EtOH exposure by activating NF-kB-dependent transcription, which may itself contribute to muscle damage. 

The transcription factor p53 induces apoptosis in various cell types in response to stress and is important for muscle homeostasis [37,38]. We used homozygous *tp53^M214K^* mutant zebrafish [28] to ask whether EtOH causes fiber detachment via p53-dependent apoptosis. There were no significant differences in the proportion of treated embryos that displayed fiber detachments (Figure 3C, *p* = 0.19) or the average number of segments per embryo with fiber detachments (Figure 3D, *p* = 0.29) in EtOH-treated, homozygous *tp53* mutants and ABs. Therefore, EtOH-induced muscle degeneration could involve pro-inflammatory NF-kB-dependent signaling, but occurs independently of p53-mediated apoptosis. 

### 3.4. EtOH-Induced Fiber Detachments Result from Sarcolemmal Instability and Failure of Muscle End Attachments

**Figure 4:**
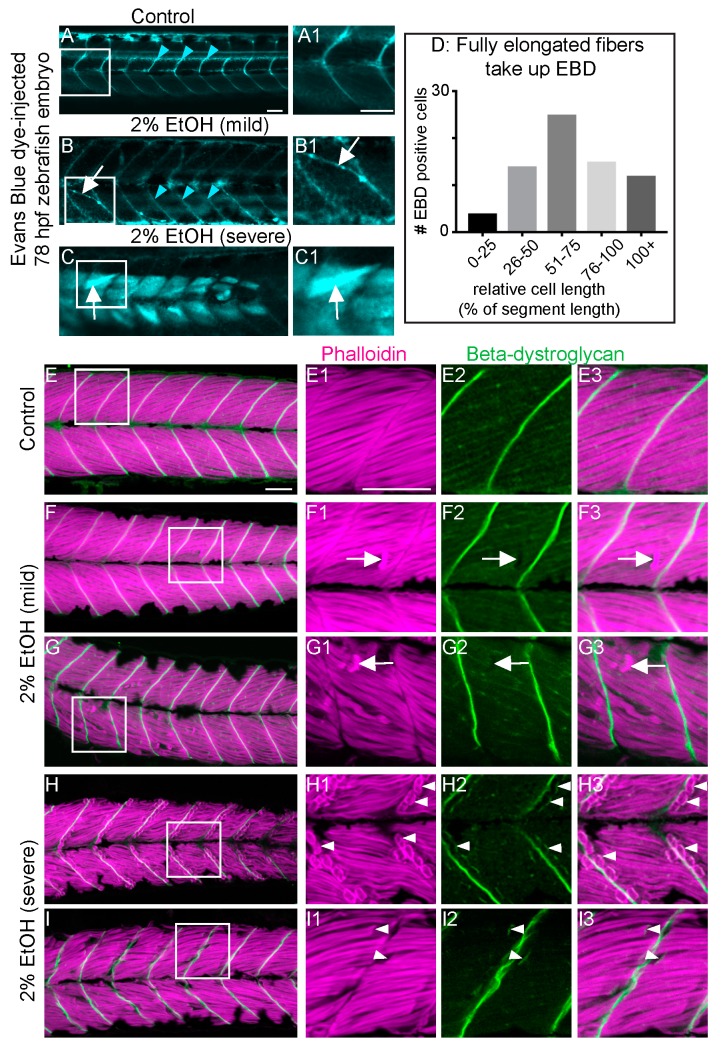
EtOH-induced fiber detachments result from sarcolemmal instability and failure of muscle end attachments. (**A**–**C1**) Anterior left, dorsal top, side-mounted, live, 78 hpf embryos treated continuously with 2% EtOH from 30 to 72 hpf, injected with 1% Evans Blue Dye (EBD) (pseudocolored blue) at 72 hpf. White boxes in A, B, and C correspond to zoomed-in, numbered panels A1, B1, and C1, respectively. (**A**–**A1**) Control embryo. EBD is in the blood stream (blue arrowheads). (**B**–**B1**) Embryo with mild fiber detachment phenotype that was induced by continuous treatment of 2% EtOH from 30 to 72 hpf. EBD is in the blood stream (blue arrowheads). EBD permeates some long muscle fibers (white arrows in B and B1). (**C**–**C1**) Embryo with severe fiber detachment phenotype that was induced by continuous treatment of 2% EtOH from 30 to 72 hpf. EBD is in the blood stream (blue arrowheads). EBD permeates long (white arrows in C and C1) and short muscle fibers. (**D**) Quantification of the relative length of EBD-penetrated myofibers. EBD penetrated myofibers ranging from short, retracted fibers to fully elongated, diagonally arrayed, fast-twitch myofibers in EtOH-treated embryos. (**E**–**I3**) Anterior left, dorsal top, side-mounted, 72 hpf embryos stained with phalloidin (pseudo-colored fuchsia) to visualize actin and anti-beta-DG antibodies (green). Lettered panels show phalloidin and beta-DG channels merged. White boxes correspond to numbered panels on the right. Panels numbered 1 show zoomed-in images of phalloidin staining only. Panels numbered 2 show zoomed-in images of beta-DG staining only. Panels numbered 3 show zoomed-in images of phalloidin and beta-DG channels merged. (**E**–**E3**) Control embryo. Fibers remained long and beta-DG is localized to the MTJ. (**F**–**G3**) Embryos treated continuously with 2% EtOH from 30 to 72 hpf showing mild fiber detachment. White arrowheads in F1–F3 and G1–G3 point to retracted fibers unaccompanied by beta-DG. (**H**–**I3**) Embryos treated continuously with 2% EtOH from 30 to 72 hpf with severe fiber detachment. White arrowheads in H1–H3 and I1–I3 point to retracted fibers that retained beta-DG at their detached ends. Scale bar is 50 micrometers.

Muscle damage in zebrafish models studied thus far can result from two distinct cellular etiologies: failure to maintain sarcolemmal integrity or failure to maintain adhesion at the MTJ external to the sarcolemma [24]. Use of cell impermeable Evans blue dye (EBD), which is only taken up by muscle when sarcolemmal integrity is compromised, and beta-Dystroglycan (beta-DG) immunohistochemistry can be used to distinguish between these two etiologies [24]. Retention of the membrane-integral protein beta-DG at the unanchored ends of retracted fibers indicates MTJ adhesion failure [39]. As shown previously [39], EBD was excluded from muscle fibers in controls (Figure 4A). EBD was observed in retracted myofibers as well as long, attached myofibers in EtOH-treated zebrafish (Figure 4B–D), suggesting that EtOH disrupts sarcolemmal integrity. Every zebrafish with muscle damage showed EBD uptake regardless of whether the phenotype was mild or severe (*n* = 30 zebrafish over 3 trials). Beta-DG robustly localized to MTJs in untreated controls (Figure 4E–E3). Beta-DG also localized to MTJs in EtOH-treated embryos that did not have muscle degeneration (not shown). Beta-DG remained at the MTJ and did not accompany retracted fibers in 19/21 EtOH-treated zebrafish with the mild phenotype (Figure 4F–G3, white arrows indicate retracted fibers unaccompanied by beta-DG). In 2/21 EtOH-treated zebrafish with the mild muscle phenotype, beta-DG remained with at least one detached muscle fiber in each embryo (not shown). Thus, the vast majority of muscle fibers in EtOH-treated zebrafish with the mild phenotype do not retain beta-DG at their unanchored ends. The opposite was observed in zebrafish with the severe phenotype. Beta-DG was observed at the unanchored ends of retracted muscle fibers in 5/6 severe zebrafish (Figure 4H–I3). Taken together, these data suggest that EtOH treatment disrupts both sarcolemmal integrity and adhesion to the MTJ, the latter is more frequently observed in cases of severe muscle degeneration.

### 3.5. Overexpression of Paxillin Does Not Rescue Fiber Detachments Caused by Loss of Sarcolemmal Integrity

**Figure 5:**
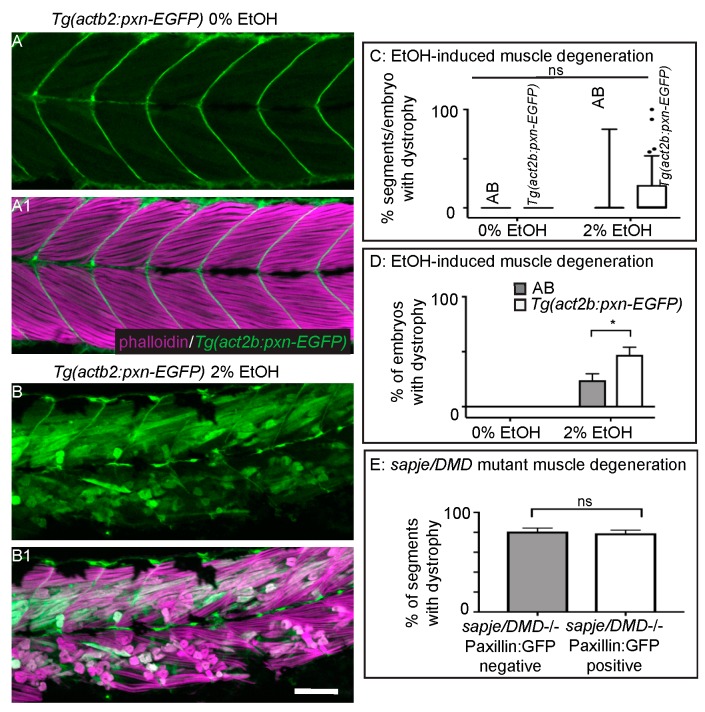
Paxillin overexpression does not rescue fiber detachments caused by loss of sarcolemmal integrity. (**A**–**B1**) Anterior left, dorsal top, side mounted, 72 hpf *Tg(act2b:pxn-EGFP)* embryos overexpressing Paxillin-EGFP (green) stained with phalloidin (pseudocolored fuchsia) to visualize actin. (**A**–**A1**) *Tg(act2b:pxn-EGFP)* control embryo. Fibers remain long and Paxillin-EGFP localizes to the MTJ. (**A**) GFP channel only. (**A1**) Phalloidin and GFP channels merged. (B–B1) *Tg(act2b:pxn-EGFP)* embryo treated continuously with 2% EtOH from 30 to 72 hpf. Fibers detach from the MTJ. (B) GFP channel only. (B1) Phalloidin and GFP channels merged. (**C**) Quantification of the percentage of imaged segments/embryo that had EtOH-induced dystrophy. Embryos that constitutively overexpressed Paxillin-EGFP (*n* = 53e) had more segments with fiber detachments compared to AB embryos (*n* = 54e) after continuous treatment with 2% EtOH. (**D**) Quantification of percent of embryos with EtOH-induced fiber detachments. Fiber detachment frequency was significantly greater in embryos that constitutively overexpressed Paxillin-EGFP compared to AB embryos after continuous treatment with 2% EtOH. (**E**) Quantification of the proportion of imaged segments/embryo with dystrophy. *sapje* mutants that constitutively overexpressed Paxillin-EGFP (white bars) (*n* = 28e) showed no significant difference in the percent of segments that contained fiber detachments compared to those that did not constitutively overexpress Paxillin-EGFP (gray bars) (*n* = 23e) (*p* = 0.11). These data suggest that Paxillin overexpression does not reduce fiber detachments due to loss of sarcolemmal integrity. Scale bars are 50 micrometers. Error bars are standard error of the mean. * *p* < 0.05.

Muscle fibers in dystrophin-null *sapje* mutant zebrafish uptake EBD prior to overt muscle degeneration [24,40]. Previous work from our lab has shown that overexpression of the cytoplasmic, Integrin adaptor protein Paxillin reduces fiber detachments caused by failure of MTJ adhesion external to the sarcolemma [26]. However, it is unknown whether Paxillin overexpression can reduce muscle degeneration that is preceded by sarcolemmal failure or whether Paxillin overexpression will benefit severe EtOH-induced muscle degeneration given that MTJ adhesion failure was observed in these cases. We thus asked whether Paxillin overexpression would reduce muscle degeneration in the contexts of EtOH exposure or in *sapje* mutant zebrafish. Transgenic zebrafish that constitutively express Paxillin (*Tg(actb2:pxn-EGFP)*) have normal muscle (Figure 5A–A1, [26]). Surprisingly, Paxillin overexpression significantly increased both the prevalence and severity of EtOH-induced muscle damage (Figure 5B–B1,C,D, *p* < 0.03 and 0.02 respectively). In contrast, Paxillin overexpression did not significantly change the phenotype of *sapje* mutants (Figure 5E, *p* = 0.11). 

### 3.6. Combining the HDAC Inhibitor TSA with EtOH Is Lethal for Zebrafish Embryos

The fact that Paxillin overexpression exacerbates muscle damage in EtOH-treated zebrafish but not *sapje* mutant zebrafish suggests that the underlying mechanisms of muscle damage are distinct despite EBD uptake in both contexts. We asked whether the HDAC-inhibitor TSA, which significantly reduces muscle degeneration in *sapje* mutant zebrafish [31],, would alter the penetrance or severity of EtOH-induced muscle damage. We found that the combination of EtOH and TSA was lethal in zebrafish (Table 1) before EtOH-induced fiber detachments occurred. Interestingly, this combination of small molecules was lethal regardless of whether the TSA was dissolved in DMSO or EtOH (data not shown). 

### 3.7. EtOH Exposure Exacerbates Muscle Degeneration in Sapje Mutant Zebrafish 

**Figure 6:**
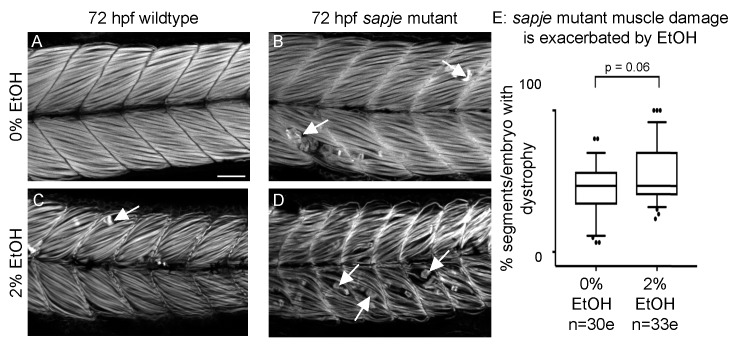
EtOH exacerbates dystrophy in *sapje* mutants. (**A**–**D**) Anterior left, dorsal top, side-mounted, 72 hpf embryos stained with phalloidin (white) to visualize actin. (**A**) Wildtype control embryo. Muscle fibers are attached to the MTJ. (**B**) *sapje* mutant control embryo. A few muscle fibers detach from the MTJ (white arrows). (**C**) Wildtype embryo treated continuously with 2% EtOH from 30 to 72 hpf. A few muscle fibers detach from the MTJ (white arrow). (**D**) *sapje* mutant embryo treated continuously with 2% EtOH from 30 to 72 hpf. Note the increased number of muscle fibers detached from the MTJ (white arrows). (**E**) Quantification of fiber detachment frequency in *sapje* mutant embryos without and with EtOH exposure. *sapje* mutant embryos that were treated continuously with 2% EtOH showed an average increase in fiber detachments per embryo (*p* = 0.06). “e” = embryo. Whiskers are 10th–90th percentiles.

Given the differences between *sapje* mutant and EtOH-treated zebrafish, we asked whether EtOH treatment would additively worsen muscle damage in *sapje* mutant zebrafish. Wildtype zebrafish not exposed to EtOH displayed normal muscle (Figure 6A). *sapje* mutants not exposed to EtOH or wildtype embryos exposed to 2% EtOH had relatively few foci of muscle damage (Figure 6B–C, white arrows). *sapje* mutants treated with 2% EtOH displayed more segments with muscle damage per embryo than either unexposed *sapje* mutants or exposed wildtypes alone (Figure 6D, white arrows). Although EtOH-treated *sapje* mutant zebrafish trended towards having more muscle degeneration, the interaction was not quite significant (Figure 6E, *p* = 0.06).

## 4. Discussion

EtOH exposure can cause a wide range of symptoms in both fetal and adult organisms. Most of what is known about these diseases, such as in FASD, centers on the neurological defects [41]. Our data support a growing body of evidence suggesting that EtOH exposure damages skeletal muscle. We show for the first time that EtOH exposure after primary muscle development (after 24 hpf) causes muscle fiber detachment and degeneration in zebrafish (Figure 1 and Figure 2). This result contrasts with the effects of EtOH exposure *during* muscle development. EtOH treatment from 8 to 24 hpf causes abnormally wide MTJs and smaller slow-twitch muscle fibers [8] but does not cause muscle degeneration. The mechanisms underlying muscle degeneration in zebrafish exposed to EtOH after 24 hpf are not known. We show that EtOH treatment results in a pro-inflammatory stress response, as indicated by NF-kB induction in skeletal muscle (Figure 3). EtOH treatment also causes sarcolemmal damage, which is visualized by uptake of EBD (Figure 4). In severely affected embryos, EtOH treatment results in failure of muscle end attachments to the ECM at the MTJ (Figure 4). We tested whether two approaches that reduce muscle damage in contexts where there is sarcolemmal damage or disrupted muscle-MTJ adhesion could reduce degeneration upon EtOH exposure. Neither of these approaches worked: TSA interacts with EtOH and is lethal, and Paxillin overexpression exacerbated muscle degeneration. Taken together, these data indicate that EtOH likely has pleiotropic effects on zebrafish skeletal muscle. 

### 4.1. Variability in the Penetrance and Severity of Muscle Damage Caused by EtOH

We report here that there is some variation in the penetrance and severity of skeletal muscle damage accompanying EtOH treatment between trials. We do not know if this variation is specific to this time point because previous reports have compiled trials. However, it has been shown that EtOH affects neurobehavioral responses differently in different strains of zebrafish [8]. Although we use the AB strain, we have not characterized the genetic variation in our zebrafish facility. Thus, one future avenue of investigation would be to determine the potential genetic modifiers that provide more resilience to EtOH treatment in some zebrafish. 

The decreasing muscle damage associated with increased age at initial EtOH exposure is likely due to either less EtOH ingestion and/or enhanced EtOH metabolism in older embryos. It was observed that the concentration of EtOH within zebrafish embryos was 2.7–4.2 fold lower in 24 hpf zebrafish and 5.8–6.2 fold less in 48 hpf zebrafish than the waterborne EtOH concentration [42]. Therefore, zebrafish may develop a barrier to EtOH as they age [42]. However, another study found the percentage of the waterborne EtOH concentration detected within embryos steadily rose from about 15–30% when embryos were exposed to the same concentration of EtOH for increasing lengths of time (i.e., 10–48 h of continuous EtOH exposure) [43]. Furthermore, phenotypes of interest are usually elicited upon exposing adult zebrafish to 0.5–1% EtOH (*v*/*v*) whereas the EtOH dose used on zebrafish embryos is usually 1.5–2.5% EtOH. Together, these observations potentially contradict the idea that zebrafish develop a barrier to EtOH as they age.

### 4.2. EtOH Treatment Damages the Muscle Sarcolemma and Disrupts Muscle Fiber-MTJ Adhesion

Muscle atrophy is a well-known symptom of many chemical or environmental insults and genetic perturbations. Previous studies showed that muscle fibers can detach before undergoing cell death via at least two distinct cellular etiologies in zebrafish models of genetic muscle diseases [24]. Here, we show that EtOH exposure can result in mild (10 or fewer segments with muscle degeneration) or severe (> 10 segments with muscle degeneration) phenotypes. The fact that muscle fibers in both phenotypic categories uptake EBD indicates that EtOH causes damage to the muscle sarcolemma regardless of phenotypic severity. In contrast, disruption of muscle fiber-MTJ adhesion correlated with phenotypic severity. Out of 21 EtOH exposed zebrafish with mild muscle damage, only two zebrafish had one or more muscle fibers each that retained beta-Dystroglycan at their terminal ends. In contrast, the vast majority of muscle fibers retained beta-Dystroglycan at their terminal ends in zebrafish with the severe muscle phenotype (5/6 zebrafish, when damage is severe it is impossible to count how many fibers have detached). It will be interesting in the future to determine the mechanisms underlying disruption of muscle cell-MTJ adhesion in zebrafish with the severe phenotype. 

### 4.3. Interaction of Environment and Muscle Disease 

One hallmark of many congenital muscular dystrophies is their phenotypic variation but the underlying causes of variation are not known. We previously showed that the human influenza A virus (IAV) damages muscle in zebrafish embryos. IAV infection significantly increases muscle degeneration and decreases survival in the zebrafish model of Duchenne Muscular Dystrophy (dystrophin-deficient *sapje* mutant zebrafish) [25]. Here we asked whether EtOH treatment similarly exacerbated muscle degeneration in *sapje* mutant zebrafish. Although we did not observe a significant interaction, there was a clear trend towards worsening of the *sapje* phenotype (Figure 6, *p* = 0.06). Taken together these data highlight the utility of the zebrafish model to integrate gene environment interactions and suggest that environmental insults may have more pleiotropic deleterious effects on muscle tissue than genetic perturbations. 

## Figures and Tables

**Table 1:** EtOH and Trichostatin A (TSA) is a Lethal Combination for Zebrafish Embryos.

Experimental Group	Total Embryos	Embryos Alive at 72 hpf	%Survival
0.66% DMSO	48	48	100
TSA (200 nM) with 0.66% DMSO	52	52	100
EtOH (2%)	50	36	72
EtOH (2%) with 0.66% DMSO	27	20	74
EtOH (2%) + TSA (200 nM) with 0.66% DMSO	54	2	3.7

## Supplementary Materials

The supplementary materials are available online http://www.mdpi.com/2221-3759/6/1/7/s1.
