# Supplementary material for: Ethanol Exposure Causes Muscle Degeneration in Zebrafish
Source: J Dev Biol. 2018 Mar 9;6(1):7. doi: 10.3390/jdb6010007 (PMC5875561; doi:10.3390/jdb6010007)
Supplement: Supplementary file 1 [file jdb-06-00007-s001.pdf]

Coffey et al. Statistical details (One-way ANOVA: multiple comparisons)

Fig 1 D

Number of families	1					
Number of comparisons per family	15					
Alpha	0.05					
Tukey's multiple comparisons test	Mean Diff.	95.00% CI of diff.	Significant?	Summary	Adjusted P Value	
0% EtOH vs. 0.5% EtOH	0	-0.0761 to 0.0761	No	ns	>0.9999	A-B
0% EtOH vs. 1% EtOH	0	-0.0761 to 0.0761	No	ns	>0.9999	A-C
0% EtOH vs. 1.5% EtOH	0	-0.0761 to 0.0761	No	ns	>0.9999	A-D
0% EtOH vs. 2% EtOH	0.04348	-0.03262 to 0.1196	No	ns	0.5751	A-E
0% EtOH vs. 2.5% EtOH	0.8841	0.808 to 0.9602	Yes	****	<0.0001	A-F
0.5% EtOH vs. 1% EtOH	0	-0.0761 to 0.0761	No	ns	>0.9999	B-C
0.5% EtOH vs. 1.5% EtOH	0	-0.0761 to 0.0761	No	ns	>0.9999	B-D
0.5% EtOH vs. 2% EtOH	0.04348	-0.03262 to 0.1196	No	ns	0.5751	B-E
0.5% EtOH vs. 2.5% EtOH	0.8841	0.808 to 0.9602	Yes	****	<0.0001	B-F
1% EtOH vs. 1.5% EtOH	0	-0.0761 to 0.0761	No	ns	>0.9999	C-D
1% EtOH vs. 2% EtOH	0.04348	-0.03262 to 0.1196	No	ns	0.5751	C-E
1% EtOH vs. 2.5% EtOH	0.8841	0.808 to 0.9602	Yes	****	<0.0001	C-F
1.5% EtOH vs. 2% EtOH	0.04348	-0.03262 to 0.1196	No	ns	0.5751	D-E
1.5% EtOH vs. 2.5% EtOH	0.8841	0.808 to 0.9602	Yes	****	<0.0001	D-F
2% EtOH vs. 2.5% EtOH	0.8406	0.7645 to 0.9167	Yes	****	<0.0001	E-F

Test details	Mean 1	Mean 2	Mean Diff.	SE of diff.	n1	n2	q	DF
0% EtOH vs. 0.5% EtOH	1	1	0	0.02658	69	69	0	408
0% EtOH vs. 1% EtOH	1	1	0	0.02658	69	69	0	408
0% EtOH vs. 1.5% EtOH	1	1	0	0.02658	69	69	0	408
0% EtOH vs. 2% EtOH	1	0.9565	0.04348	0.02658	69	69	2.314	408
0% EtOH vs. 2.5% EtOH	1	0.1159	0.8841	0.02658	69	69	47.04	408
0.5% EtOH vs. 1% EtOH	1	1	0	0.02658	69	69	0	408
0.5% EtOH vs. 1.5% EtOH	1	1	0	0.02658	69	69	0	408
0.5% EtOH vs. 2% EtOH	1	0.9565	0.04348	0.02658	69	69	2.314	408
0.5% EtOH vs. 2.5% EtOH	1	0.1159	0.8841	0.02658	69	69	47.04	408
1% EtOH vs. 1.5% EtOH	1	1	0	0.02658	69	69	0	408
1% EtOH vs. 2% EtOH	1	0.9565	0.04348	0.02658	69	69	2.314	408
1% EtOH vs. 2.5% EtOH	1	0.1159	0.8841	0.02658	69	69	47.04	408
1.5% EtOH vs. 2% EtOH	1	0.9565	0.04348	0.02658	69	69	2.314	408
1.5% EtOH vs. 2.5% EtOH	1	0.1159	0.8841	0.02658	69	69	47.04	408
2% EtOH vs. 2.5% EtOH	0.9565	0.1159	0.8406	0.02658	69	69	44.73	408

Fig 1 L

Number of families	1					
Number of comparisons per family	15					
Alpha	0.05					
Tukey's multiple comparisons test	Mean Diff.	95.00% CI of diff.	Significant?	Summary	Adjusted P Value	
0 vs. 0.5	-0.2933	-0.5218 to -0.06486	Yes	**	0.0036	A-B
0 vs. 1	-0.1644	-0.3941 to 0.06537	No	ns	0.3172	A-C
0 vs. 1.5	-0.3738	-0.5879 to -0.1598	Yes	****	<0.0001	A-D
0 vs. 2	-0.4727	-0.6858 to -0.2597	Yes	****	<0.0001	A-E
0 vs. 2.5	-0.5714	-0.8235 to -0.3194	Yes	****	<0.0001	A-F
0.5 vs. 1	0.1289	-0.07922 to 0.3371	No	ns	0.4845	B-C
0.5 vs. 1.5	-0.0805	-0.2712 to 0.1102	No	ns	0.8328	B-D
0.5 vs. 2	-0.1794	-0.369 to 0.0102	No	ns	0.0756	B-E
0.5 vs. 2.5	-0.2781	-0.5107 to -0.04553	Yes	**	0.0088	B-F
1 vs. 1.5	-0.2094	-0.4016 to -0.01725	Yes	*	0.0236	C-D
1 vs. 2	-0.3083	-0.4995 to -0.1172	Yes	****	<0.0001	C-E
1 vs. 2.5	-0.407	-0.6409 to -0.1732	Yes	****	<0.0001	C-F
1.5 vs. 2	-0.0989	-0.2708 to 0.07302	No	ns	0.5682	D-E
1.5 vs. 2.5	-0.1976	-0.416 to 0.0208	No	ns	0.1019	D-F
2 vs. 2.5	-0.0987	-0.3162 to 0.1188	No	ns	0.7856	E-F

Test details	Mean 1	Mean 2	Mean Diff.	SE of diff.	n1	n2	q	DF
0 vs. 0.5	0	0.2933	-0.2933	0.07984	52	75	5.196	460
0 vs. 1	0	0.1644	-0.1644	0.08028	52	73	2.896	460
0 vs. 1.5	0	0.3738	-0.3738	0.07479	52	107	7.069	460
0 vs. 2	0	0.4727	-0.4727	0.07446	52	110	8.979	460
0 vs. 2.5	0	0.5714	-0.5714	0.08808	52	49	9.174	460
0.5 vs. 1	0.2933	0.1644	0.1289	0.07274	75	73	2.507	460
0.5 vs. 1.5	0.2933	0.3738	-0.0805	0.06663	75	107	1.709	460
0.5 vs. 2	0.2933	0.4727	-0.1794	0.06625	75	110	3.829	460
0.5 vs. 2.5	0.2933	0.5714	-0.2781	0.08127	75	49	4.839	460
1 vs. 1.5	0.1644	0.3738	-0.2094	0.06716	73	107	4.41	460
1 vs. 2	0.1644	0.4727	-0.3083	0.06679	73	110	6.529	460
1 vs. 2.5	0.1644	0.5714	-0.407	0.08171	73	49	7.045	460
1.5 vs. 2	0.3738	0.4727	-0.0989	0.06007	107	110	2.328	460
1.5 vs. 2.5	0.3738	0.5714	-0.1976	0.07631	107	49	3.662	460
2 vs. 2.5	0.4727	0.5714	-0.0987	0.07599	110	49	1.837	460

Fig 1 M

Number of families	1					
Number of comparisons per family	15					
Alpha	0.05					
Tukey's multiple comparisons test	Mean Diff.	95.00% CI of diff.	Significant?	Summary	Adjusted P Value	
0 vs. 0.5	-0.09107	-0.2524 to 0.07028	No	ns	0.5889	A-B
0 vs. 1	-0.02973	-0.192 to 0.1325	No	ns	0.9952	A-C
0 vs. 1.5	-0.1921	-0.3432 to -0.04091	Yes	**	0.0041	A-D
0 vs. 2	-0.314	-0.4645 to -0.1635	Yes	****	<0.0001	A-E
0 vs. 2.5	-0.4386	-0.6166 to -0.2606	Yes	****	<0.0001	A-F
0.5 vs. 1	0.06134	-0.08566 to 0.2083	No	ns	0.8396	B-C
0.5 vs. 1.5	-0.101	-0.2356 to 0.03366	No	ns	0.2654	B-D
0.5 vs. 2	-0.2229	-0.3568 to -0.08904	Yes	****	<0.0001	B-E
0.5 vs. 2.5	-0.3475	-0.5117 to -0.1833	Yes	****	<0.0001	B-F
1 vs. 1.5	-0.1623	-0.2981 to -0.0266	Yes	**	0.0088	C-D
1 vs. 2	-0.2843	-0.4192 to -0.1493	Yes	****	<0.0001	C-E
1 vs. 2.5	-0.4088	-0.574 to -0.2437	Yes	****	<0.0001	C-F
1.5 vs. 2	-0.1219	-0.2433 to -0.0005409	Yes	*	0.0483	D-E
1.5 vs. 2.5	-0.2465	-0.4007 to -0.09229	Yes	****	<0.0001	D-F
2 vs. 2.5	-0.1246	-0.2781 to 0.02899	No	ns	0.1875	E-F

Test details	Mean 1	Mean 2	Mean Diff.	SE of diff.	n1	n2	q	DF
0 vs. 0.5	0	0.09107	-0.09107	0.05638	52	75	2.284	460
0 vs. 1	0	0.02973	-0.02973	0.05669	52	73	0.7415	460
0 vs. 1.5	0	0.1921	-0.1921	0.05282	52	107	5.143	460
0 vs. 2	0	0.314	-0.314	0.05258	52	110	8.446	460
0 vs. 2.5	0	0.4386	-0.4386	0.0622	52	49	9.971	460
0.5 vs. 1	0.09107	0.02973	0.06134	0.05137	75	73	1.689	460
0.5 vs. 1.5	0.09107	0.1921	-0.101	0.04705	75	107	3.035	460
0.5 vs. 2	0.09107	0.314	-0.2229	0.04679	75	110	6.739	460
0.5 vs. 2.5	0.09107	0.4386	-0.3475	0.05739	75	49	8.563	460
1 vs. 1.5	0.02973	0.1921	-0.1623	0.04743	73	107	4.84	460
1 vs. 2	0.02973	0.314	-0.2843	0.04717	73	110	8.524	460
1 vs. 2.5	0.02973	0.4386	-0.4088	0.0577	73	49	10.02	460
1.5 vs. 2	0.1921	0.314	-0.1219	0.04242	107	110	4.065	460
1.5 vs. 2.5	0.1921	0.4386	-0.2465	0.05389	107	49	6.469	460
2 vs. 2.5	0.314	0.4386	-0.1246	0.05366	110	49	3.283	460

Fig 1 N

Number of families	1					
Number of comparisons per family	15					
Alpha	0.05					
Tukey's multiple comparisons test	Mean Diff.	95.00% CI of diff.	Significant?	Summary	Adjusted P Value	
Trial 1 200 proof vs. Trial 2 200 proof	-0.2317	-0.5109 to 0.04746	No	ns	0.1655	A-B
Trial 1 200 proof vs. Trial 3 200 proof	-0.2647	-0.567 to 0.03765	No	ns	0.1236	A-C
Trial 1 200 proof vs. Trial 1 190 proof	-0.4386	-0.7117 to -0.1655	Yes	****	<0.0001	A-D
Trial 1 200 proof vs. Trial 2 190 proof	-0.2568	-0.5245 to 0.01093	No	ns	0.0684	A-E
Trial 1 200 proof vs. Trial 3 190 proof	0.01038	-0.228 to 0.2488	No	ns	>0.9999	A-F
Trial 2 200 proof vs. Trial 3 200 proof	-0.03298	-0.3747 to 0.3087	No	ns	0.9998	B-C
Trial 2 200 proof vs. Trial 1 190 proof	-0.2069	-0.5231 to 0.1093	No	ns	0.4163	B-D
Trial 2 200 proof vs. Trial 2 190 proof	-0.02508	-0.3366 to 0.2864	No	ns	>0.9999	B-E
Trial 2 200 proof vs. Trial 3 190 proof	0.2421	-0.04466 to 0.5288	No	ns	0.1514	B-F
Trial 3 200 proof vs. Trial 1 190 proof	-0.1739	-0.5107 to 0.1629	No	ns	0.6744	C-D
Trial 3 200 proof vs. Trial 2 190 proof	0.007905	-0.3245 to 0.3403	No	ns	>0.9999	C-E
Trial 3 200 proof vs. Trial 3 190 proof	0.2751	-0.03428 to 0.5844	No	ns	0.1127	C-F
Trial 1 190 proof vs. Trial 2 190 proof	0.1818	-0.1243 to 0.4879	No	ns	0.5281	D-E
Trial 1 190 proof vs. Trial 3 190 proof	0.449	0.1681 to 0.7298	Yes	***	0.0001	D-F
Trial 2 190 proof vs. Trial 3 190 proof	0.2672	-0.008444 to 0.5428	No	ns	0.0633	E-F

Test details	Mean 1	Mean 2	Mean Diff.	SE of diff.	n1	n2	q	DF
Trial 1 200 proof vs. Trial 2 200 proof	0.5614	0.7931	-0.2317	0.09708	57	29	3.375	216
Trial 1 200 proof vs. Trial 3 200 proof	0.5614	0.8261	-0.2647	0.1051	57	23	3.56	216
Trial 1 200 proof vs. Trial 1 190 proof	0.5614	1	-0.4386	0.09498	57	31	6.53	216
Trial 1 200 proof vs. Trial 2 190 proof	0.5614	0.8182	-0.2568	0.0931	57	33	3.901	216
Trial 1 200 proof vs. Trial 3 190 proof	0.5614	0.551	0.01038	0.08292	57	49	0.1771	216
Trial 2 200 proof vs. Trial 3 200 proof	0.7931	0.8261	-0.03298	0.1188	29	23	0.3925	216
Trial 2 200 proof vs. Trial 1 190 proof	0.7931	1	-0.2069	0.11	29	31	2.661	216
Trial 2 200 proof vs. Trial 2 190 proof	0.7931	0.8182	-0.02508	0.1083	29	33	0.3274	216
Trial 2 200 proof vs. Trial 3 190 proof	0.7931	0.551	0.2421	0.09972	29	49	3.433	216
Trial 3 200 proof vs. Trial 1 190 proof	0.8261	1	-0.1739	0.1171	23	31	2.1	216
Trial 3 200 proof vs. Trial 2 190 proof	0.8261	0.8182	0.007905	0.1156	23	33	0.0967	216
Trial 3 200 proof vs. Trial 3 190 proof	0.8261	0.551	0.2751	0.1076	23	49	3.616	216
Trial 1 190 proof vs. Trial 2 190 proof	1	0.8182	0.1818	0.1065	31	33	2.415	216
Trial 1 190 proof vs. Trial 3 190 proof	1	0.551	0.449	0.09768	31	49	6.501	216
Trial 2 190 proof vs. Trial 3 190 proof	0.8182	0.551	0.2672	0.09585	33	49	3.942	216

Fig 1 O

Number of families	1					
Number of comparisons per family	15					
Alpha	0.05					
Tukey's multiple comparisons test	Mean Diff.	95.00% CI of diff.	Significant?	Summary	Adjusted P Value	
Trial 1 200 proof vs. Trial 2 200 proof	-37.86	-59.66 to -16.07	Yes	****	<0.0001	A-B
Trial 1 200 proof vs. Trial 3 200 proof	-23.98	-47.59 to -0.3813	Yes	*	0.0440	A-C
Trial 1 200 proof vs. Trial 1 190 proof	1.156	-20.17 to 22.48	No	ns	>0.9999	A-D
Trial 1 200 proof vs. Trial 2 190 proof	0.5965	-20.3 to 21.5	No	ns	>0.9999	A-E
Trial 1 200 proof vs. Trial 3 190 proof	23.24	4.623 to 41.85	Yes	**	0.0054	A-F
Trial 2 200 proof vs. Trial 3 200 proof	13.88	-12.8 to 40.56	No	ns	0.6672	B-C
Trial 2 200 proof vs. Trial 1 190 proof	39.02	14.34 to 63.7	Yes	***	0.0001	B-D
Trial 2 200 proof vs. Trial 2 190 proof	38.46	14.14 to 62.78	Yes	***	0.0001	B-E
Trial 2 200 proof vs. Trial 3 190 proof	61.1	38.71 to 83.48	Yes	****	<0.0001	B-F
Trial 3 200 proof vs. Trial 1 190 proof	25.14	-1.155 to 51.43	No	ns	0.0700	C-D
Trial 3 200 proof vs. Trial 2 190 proof	24.58	-1.373 to 50.53	No	ns	0.0748	C-E
Trial 3 200 proof vs. Trial 3 190 proof	47.22	23.07 to 71.37	Yes	****	<0.0001	C-F
Trial 1 190 proof vs. Trial 2 190 proof	-0.5591	-24.46 to 23.34	No	ns	>0.9999	D-E
Trial 1 190 proof vs. Trial 3 190 proof	22.08	0.1538 to 44.01	Yes	*	0.0473	D-F
Trial 2 190 proof vs. Trial 3 190 proof	22.64	1.124 to 44.16	Yes	*	0.0328	E-F

Test details	Mean 1	Mean 2	Mean Diff.	SE of diff.	n1	n2	q	DF
Trial 1 200 proof vs. Trial 2 200 proof	31.93	69.79	-37.86	7.579	57	29	7.065	216
Trial 1 200 proof vs. Trial 3 200 proof	31.93	55.91	-23.98	8.208	57	23	4.132	216
Trial 1 200 proof vs. Trial 1 190 proof	31.93	30.77	1.156	7.415	57	31	0.2204	216
Trial 1 200 proof vs. Trial 2 190 proof	31.93	31.33	0.5965	7.268	57	33	0.1161	216
Trial 1 200 proof vs. Trial 3 190 proof	31.93	8.694	23.24	6.473	57	49	5.077	216
Trial 2 200 proof vs. Trial 3 200 proof	69.79	55.91	13.88	9.278	29	23	2.116	216
Trial 2 200 proof vs. Trial 1 190 proof	69.79	30.77	39.02	8.584	29	31	6.428	216
Trial 2 200 proof vs. Trial 2 190 proof	69.79	31.33	38.46	8.457	29	33	6.431	216
Trial 2 200 proof vs. Trial 3 190 proof	69.79	8.694	61.1	7.785	29	49	11.1	216
Trial 3 200 proof vs. Trial 1 190 proof	55.91	30.77	25.14	9.144	23	31	3.888	216
Trial 3 200 proof vs. Trial 2 190 proof	55.91	31.33	24.58	9.025	23	33	3.851	216
Trial 3 200 proof vs. Trial 3 190 proof	55.91	8.694	47.22	8.398	23	49	7.951	216
Trial 1 190 proof vs. Trial 2 190 proof	30.77	31.33	-0.5591	8.311	31	33	0.09515	216
Trial 1 190 proof vs. Trial 3 190 proof	30.77	8.694	22.08	7.625	31	49	4.095	216
Trial 2 190 proof vs. Trial 3 190 proof	31.33	8.694	22.64	7.482	33	49	4.279	216

Fig 5 F

Number of families	1							
Number of comparisons per family	6							
Alpha	0.05							
Tukey's multiple comparisons test	Mean Diff.	95.00% CI of diff.	Significant?	Summary	Adjusted P Value			
AB control 0% vs. AB control 2%	-0.2407	-0.5949 to 0.1134	No	ns	0.2928	A-B		
AB control 0% vs. bactin paxillin 0%	0	-0.4531 to 0.4531	No	ns	>0.9999	A-C		
AB control 0% vs. bactin paxillin 2%	-0.4717	-0.8265 to -0.1169	Yes	**	0.0040	A-D		
AB control 2% vs. bactin paxillin 0%	0.2407	-0.1134 to 0.5949	No	ns	0.2928	B-C		
AB control 2% vs. bactin paxillin 2%	-0.231	-0.4455 to -0.01638	Yes	*	0.0296	B-D		
bactin paxillin 0% vs. bactin paxillin 2%	-0.4717	-0.8265 to -0.1169	Yes	**	0.0040	C-D		
Test details	Mean 1	Mean 2	Mean Diff.	SE of diff.	n1	n2	q	DF
AB control 0% vs. AB control 2%	0	0.2407	-0.2407	0.136	12	54	2.503	127
AB control 0% vs. bactin paxillin 0%	0	0	0	0.174	12	12	0	127
AB control 0% vs. bactin paxillin 2%	0	0.4717	-0.4717	0.1363	12	53	4.895	127
AB control 2% vs. bactin paxillin 0%	0.2407	0	0.2407	0.136	54	12	2.503	127
AB control 2% vs. bactin paxillin 2%	0.2407	0.4717	-0.231	0.08242	54	53	3.963	127
bactin paxillin 0% vs. bactin paxillin 2%	0	0.4717	-0.4717	0.1363	12	53	4.895	127
